# Supplementary material for: CN133, a Novel Brain-Penetrating Histone Deacetylase Inhibitor, Hampers Tumor Growth in Patient-Derived Pediatric Posterior Fossa Ependymoma Models
Source: Cancers (Basel). 2020 Jul 16;12(7):1922. doi: 10.3390/cancers12071922 (PMC7409080; doi:10.3390/cancers12071922)
Supplement: Supplementary file 1 [file cancers-12-01922-s001.zip › cancers-863779-supplementary final/cancers-863779-supplementary final.docx]

Supplementary Materials

CN133, a Novel Brain-Penetrating Histone Deacetylase Inhibitor, Hampers Tumor Growth in Patient-Derived Pediatric Posterior Fossa Ependymoma

Roberta Antonelli, Carlos Jiménez, Misha Riley, Tiziana Servidei, Riccardo Riccardi, Aroa Soriano, Josep Roma, Elena Martínez-Saez, Maurizio Martini, Antonio Ruggiero, Lucas Moreno,
Josep Sánchez de Toledo, Soledad Gallego, Jordi Bové, Jacob M. Hooker and Miguel F. Segura

**Figure S1.** Chemical structure and inhibition activity of brain-penetrating HDACi. (**a**) Chemical structure of the indicated compounds. (**b**) Concentration of inhibitor necessary to halve the acetylation of the indicated histones (IC50).

**Figure S2.** CN133 and CN147 induce PARP cleavage and Caspase 3 activation in EPN cell lines. (**a**) Representative western blot of the indicated proteins in cell lysates of EPP cells treated with either 1 μM CN133, CN147 or vehicle (DMSO) for the indicated times. Uncropped Blots of Figure S2a are shown in Figure S9. (**b,c**) Graphs showing the quantification of cleaved PARP and active caspase-3 normalized to actin levels. Each graph represents the average of three independent experiments ± SEM, ** *p* < 0.01; *** *p* < 0.001.


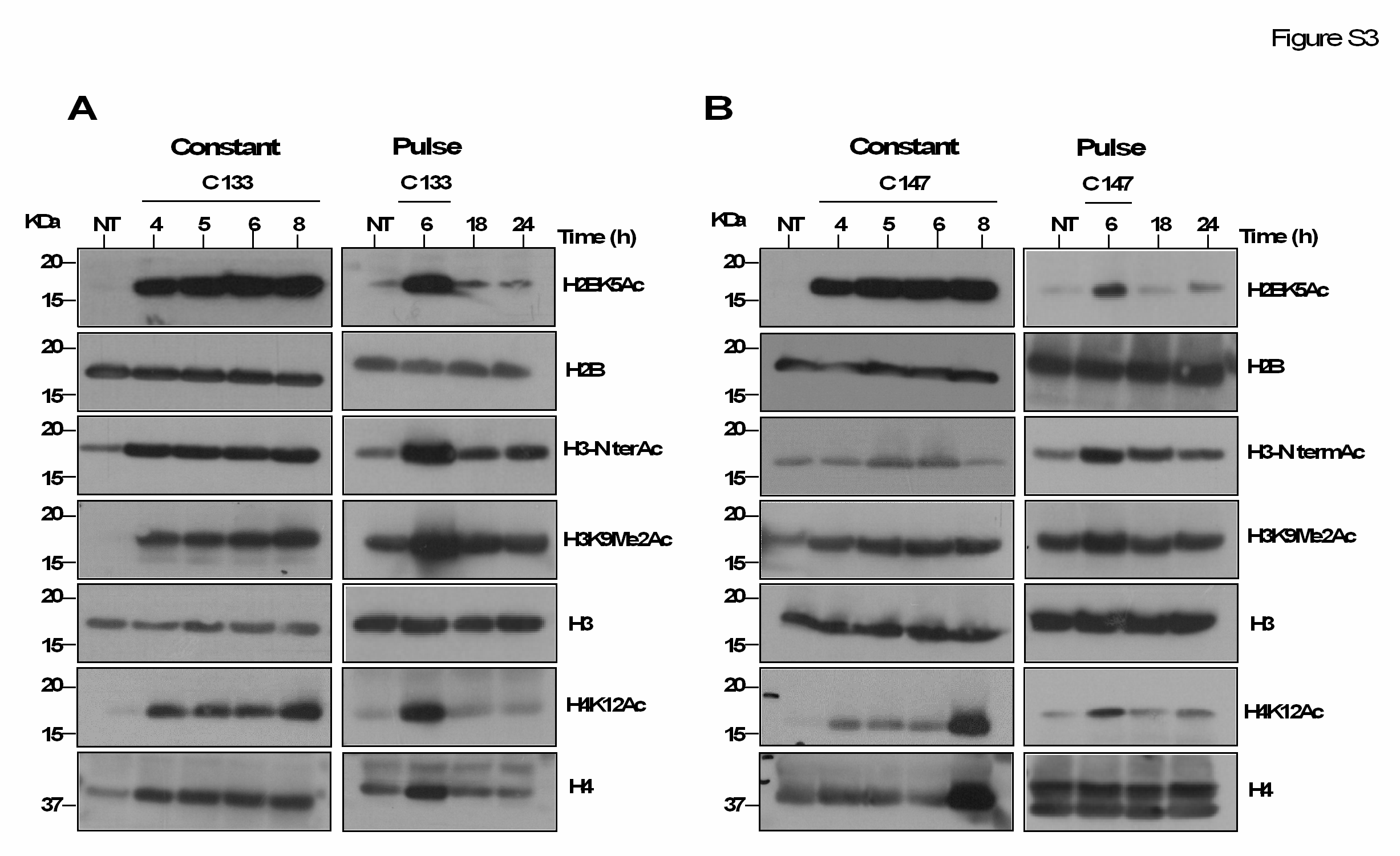


**Figure S3.** Time course of histone acetylation after constant or pulse exposure to CN133 or CN147. Western blot of histone-acetylation marks in EPP cells treated with vehicle (NT) or with 1μM of CN133 (**A**) or CN147 (**B**) for up to 8 h (Constant) or for 6 h, washed, and further incubated for up to 18 h/24 h in inhibitor-free media (Pulse). Total H2B, H3 and H4 histone levels were probed as loading control. Header lines indicates the duration of inhibitor exposure. Uncropped Blots of Figure S3 are shown in Figure S9

**Figure S4.** Monitoring of EPP tumors growth in vivo. Luciferase reporter gene-transduced EPP cell lines were implanted in the cerebellum of immunodeficient mice. Tumor growth was followed by IVIS imaging. The period while the detected signal was equal or lower than the one captured at the time of injection was considered “engraftment phase”. The phase in which the detected signal was higher in two consecutive measures was considered the “exponential growth phase”.

**Figure S5.** CN133 transcriptional analysis. (**a**) Principal component analysis showed segregation of distinct expression profiles for EPN treated with C133 or vehicle (**b**) Gene set enrichment analysis GSEA performed on genes differentially expressed in presence or absence of CN133 treatment on EPP cell line.

**Figure S6.** HDAC expression in human ependymoma samples versus healthy tissues. Data mining of HDAC1, HDAC2, HDAC3 and HDAC4 mRNA expression comparing whole brain and cerebellum (GSE11882, n = 172 and GSE = 3526, n = 9) compared with the distinct ependymoma molecular subgroups (GSE64415, n = 209).

**Figure S7.** CN133 is able to regulate p21 in a p53-independent mechanism**.** Uncropped Blots of Figure S7 are shown in Figure S9 (**a**) Western blot of PARP, P53, P21 and H2BK5Ac in EPP cells (left) and EPV cells (right) treated with 1 μM of CN133. Actin was used as a loading control. (**b**) Western blot of PARP, P53, P21 and H2BK5Ac after downregulation of P53 level and treated with 1 μM of CN133 in EPP. Actin was used as a loading control. (**c**) Western blot of PARP, P53, P21 and H2BK5Ac in p53 mut (SK-N-BE(2)) and p 53 wt (SH-SY5Y) Neuroblastoma cell lines treated with 1 μM of CN133.

**Figure S8.** Classification of the source of ependymoma cell lines**.** (**a**) Hematoxylin and eosin (H&E) staining of the original tumors where EPP (Patient 1) and EPV (Patient 2) were derived. (**b**) H3K27Me3 immunostaining of patient 1 and patient 2 tumours (upper panels). Lower panels show EPN tissue sections used as negative and positive control. Scale bars, 100 µM. (**c**) Quantitative real-time PCR (RT-qPCR) using primers for the indicated genes differentially expressed between PF-EPN-A and PF-EPN-B tumors.

Table S1. List of epigenetic drugs

| **Compound** | **Putative Target** | **Protein Family** |
| --- | --- | --- |
| **UNC1999** | EZH2 | Methyltransferase |
| **UNC0642** | G9a, GLP | Methyltransferase |
| **BAY 598** | SMYD2 | Methyltransferase |
| **GSK864** | Isocitrate dehydrogenase 1 (IDH1) | Dehydrogenase |
| **GSK484** | Protein arginine deiminase PAD4 | Arginina deiminase |
| **GSK2801** | BAZ2-icr BZA2A/B | Bromodomain |
| **TP-472** | BRD9/7 | Bromodomain |
| **JQ1** | BET | Bromodomain |
| **Tubastatin A** | HDAC 6 | Histone deacetylase |
| **Vorinostat (SAHA)** | Pan HDAC | Histone deacetylase |
| **5'-Aza** | DNMT1 | Dna methylating |

**Table S2.** List of antibodies for Western blot analyses

| **Primary Antibody** | | | |
| --- | --- | --- | --- |
| **Antibody** | **Dilution** | **Supplier** | **Reference** |
| ATF3 | 1:500 | CST | #33593 |
| ATF4 | 1:500 | CST | #11815 |
| ATF6 | 1:1000 | CST | #65880 |
| BIM | 1:1000 | CST | #2933 |
| Caspase-3 | 1:2000 | CST | #9662 |
| Caspase-3 cleaved | 1:500 | CST | #9664 |
| CHOP | 1:1000 | CST | #2895 |
| CXCR4 | 1:500 | Sigma-Aldrich | #8352 |
| Histone H3 | 1:10000 | Millipore | #05-928 |
| Histone H3K27A | 1:10000 | Millipore | #17-683 |
| Histone H2BK5Ac | 1:5000 | CST | #12799 |
| Histone H2B | 1:5000 | CST | #12364 |
| Histone H4 | 1:5000 | CST | #13919 |
| H3K9Me2Ac | 1:5000 | CST | #9649 |
| H4K12Ac | 1:5000 | CST | #2594 |
| HIF-α | 1:1000 | CST | #14179 |
| HMOX1 | 1:500 | SCBT | #136960 |
| Noxa | 1:1000 | Millipore | #114C307 |
| p-AKT^ser473^ | 1:500 | CST | #9271 |
| P21 | 1:2000 | CST | #2947 |
| p-S6 | 1:10000 | CST | #4858 |
| PARP | 1:2000 | CST | #9542 |
| PERK | 1:5000 | SCBT | #S377400 |
| XIAP | 1:3000 | BD | #610762 |
| S6 | 1:1000 | CST | #2217 |
| XIAP | 1:1000 | BD | #610762 |
| Secondary antibodies |  |  |  |
| Anti-Rabbit IgG-Peroxidase | 1:10000 | Sigma-Aldrich | #A0545 |
| Anti-Mouse IgG-Peroxidase | 1:10000 | Sigma-Aldrich | #A9044 |
| Anti-Sheep IgG-Peroxidase | 1:10000 | Sigma-Aldrich | #A3415 |

Suppliers: CST (Cell Signaling Technologies, Beverly MA, USA), SCBT (Santa Cruz Biotechnology, Santa Cruz, CA, USA), Abcam (Cambridge, UK), Merck Millipore (Billerica, MA, USA), MRC PPU Reagents and Services (Dundee, UK), Sigma-Aldrich (St. Louis, MO, USA).

**Table S3.** List of Real time PCR primers

| Gene | Sequences | | Insert Size (bp*) |
| --- | --- | --- | --- |
| SERPINE1 | Forward: | GCAACGTGGTTTTCTCACCC | 128 |
|  | Reverse: | GGCCATGCCCTTGTCATCAA |  |
| CXCR4 | Forward: | GTCACTATGGGAAAAGATGGGGA | 74 |
|  | Reverse: | AGTAGTGGGCTAAGGGCACA |  |
| EFNA3 | Forward: | ACAGCCCCATCAAGTTCTCG | 105 |
|  | Reverse: | GAGTGGGCGTGGAGATGTAG |  |
| EGRI1 | Forward: | ACAGCAACCTTTTCTCCCAG | 158 |
|  | Reverse: | CCAATAGACCTTCCACTCCAG |  |
| HMOX1 | Forward: | TCTTCACCTTCCCCAACATTG | 190 |
|  | Reverse: | CTCTGGTCCTTGGTGTCATG |  |
| GSKIP | Forward: | GCGCGCAGAATGGAAACAG | 122 |
|  | Reverse: | TCAGCTTCGAGCCTCATGTC |  |
| GADD45B | Forward: | GAAGATGCAGACGGTGACC | 193 |
|  | Reverse: | GATGAGCGTGAAGTGGATTTG |  |
| HAS1 | Forward: | CTTGTCAGAGCTACTTCCACTG | 141 |
|  | Reverse: | CGGTCATCCCCAAAAGTACAG |  |
| LRRC4b | Forward: | GCCACCTCTCTCCCCATTC | 170 |
|  | Reverse: | ACGAACCTCGCTCTTCAAG |  |

*bp: base pairs.

| 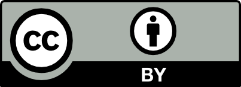 | © 2020 by the authors. Licensee MDPI, Basel, Switzerland. This article is an open access article distributed under the terms and conditions of the Creative Commons Attribution (CC BY) license (http://creativecommons.org/licenses/by/4.0/). |
| --- | --- |
