# Supplementary figures and images for: CN133, a Novel Brain-Penetrating Histone Deacetylase Inhibitor, Hampers Tumor Growth in Patient-Derived Pediatric Posterior Fossa Ependymoma Models
Source: Cancers (Basel). 2020 Jul 16;12(7):1922. doi: 10.3390/cancers12071922 (PMC7409080; doi:10.3390/cancers12071922)

Uncropped western blot Figure 1

Figure 1 C

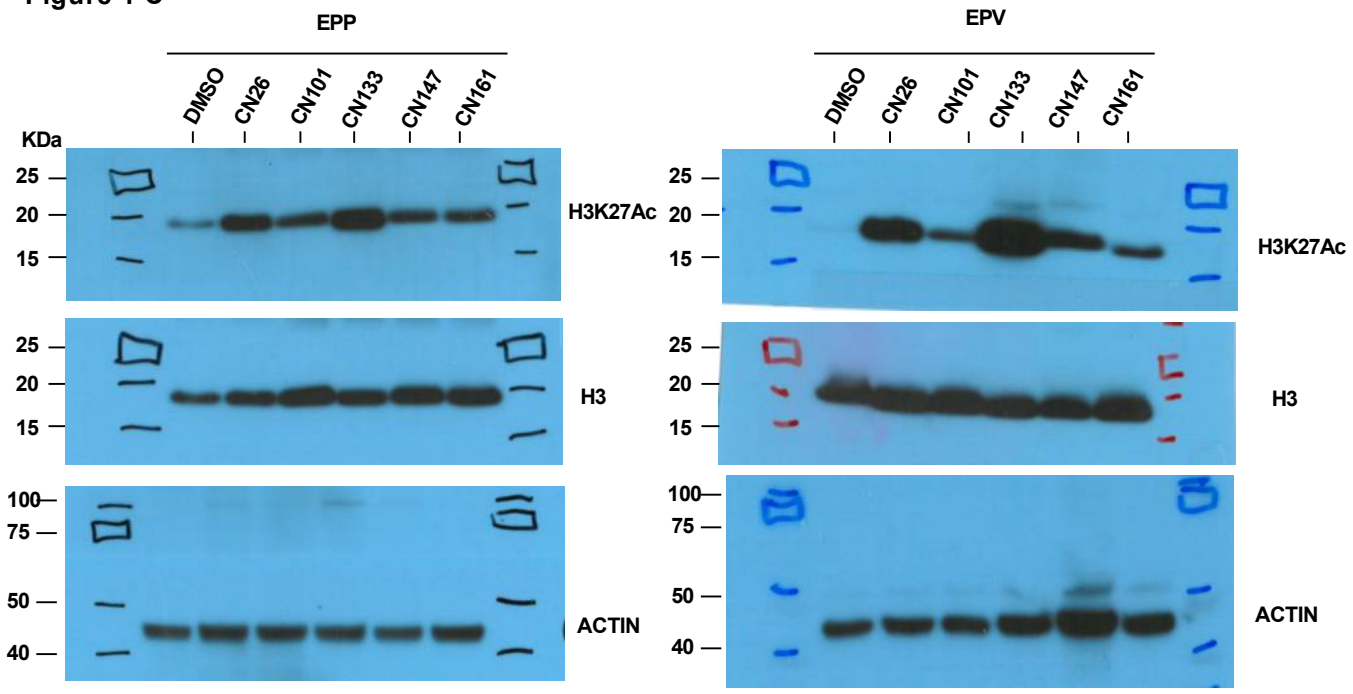

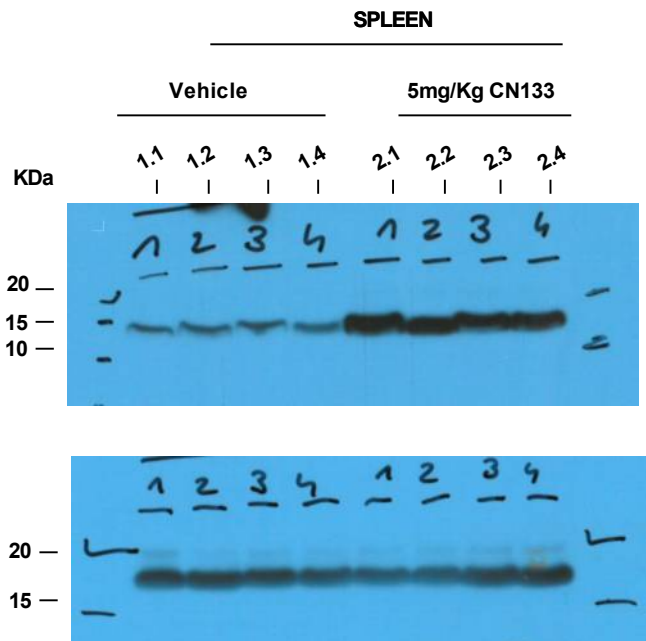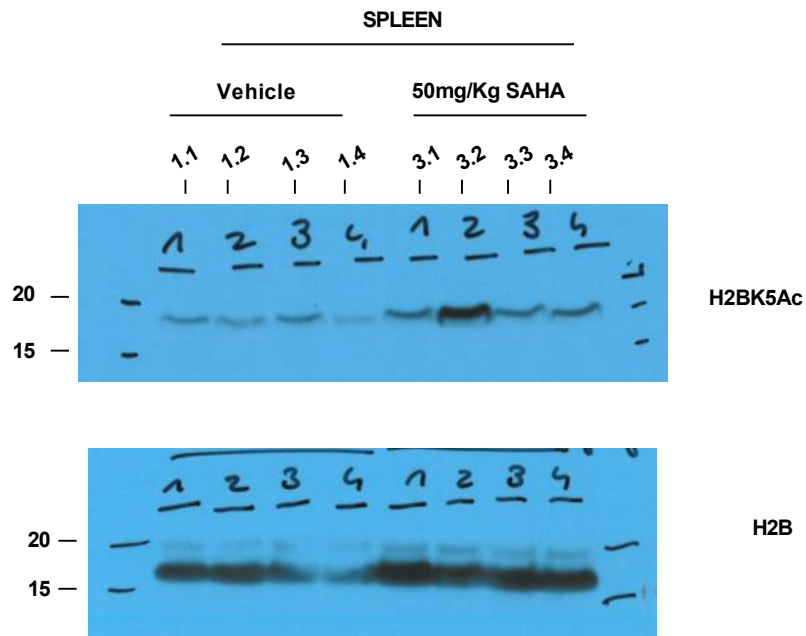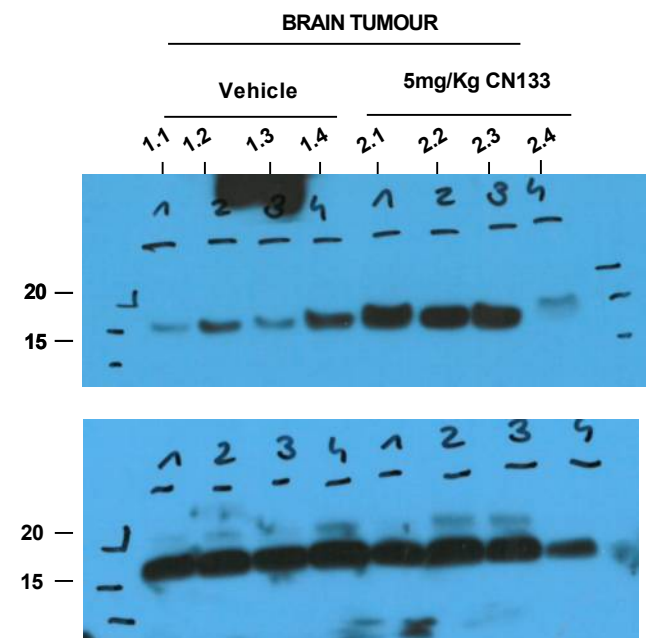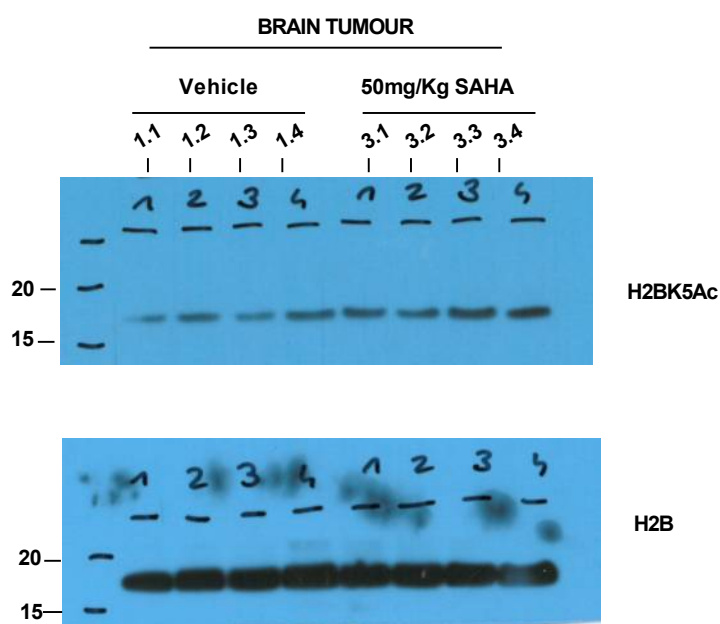

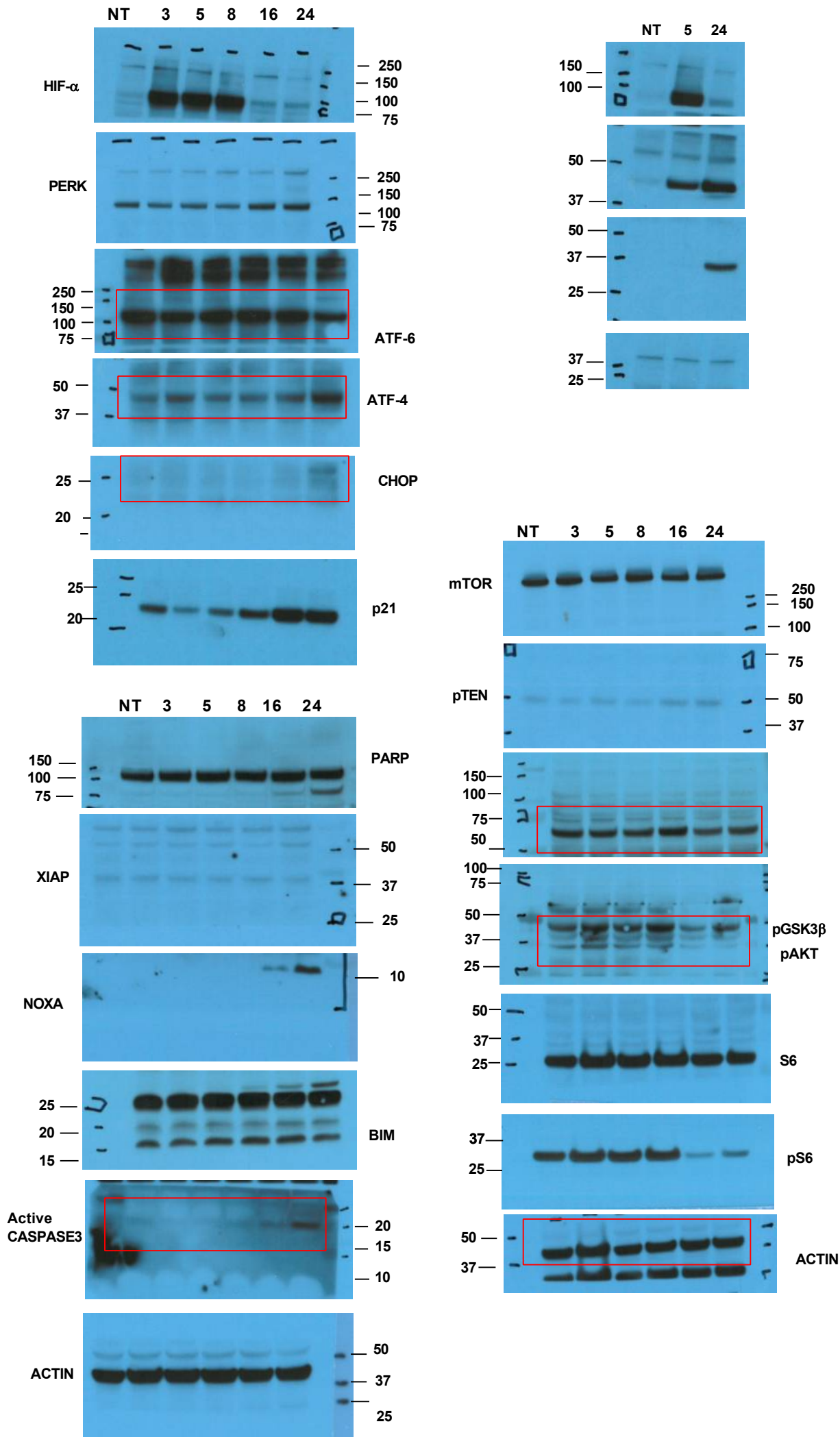

Figure S 2

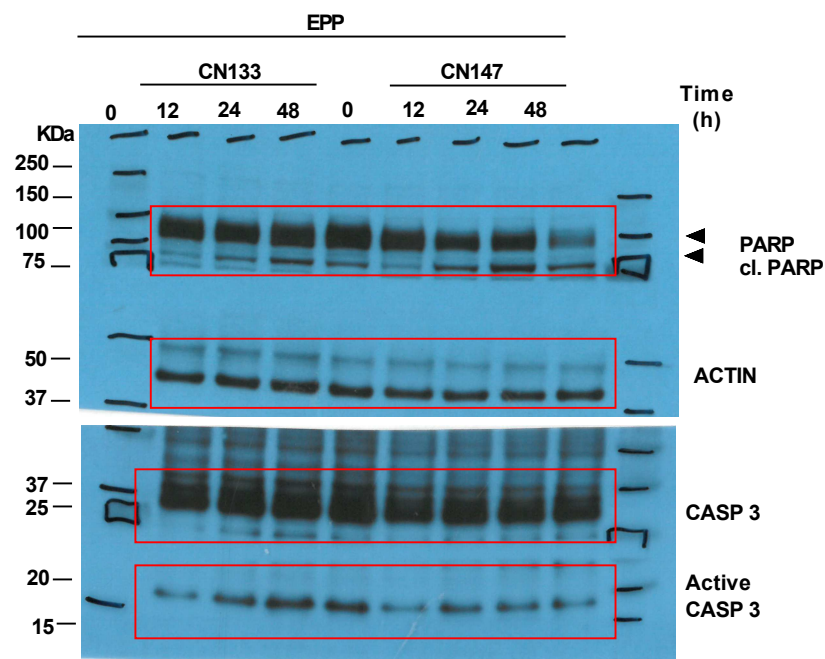

**A**

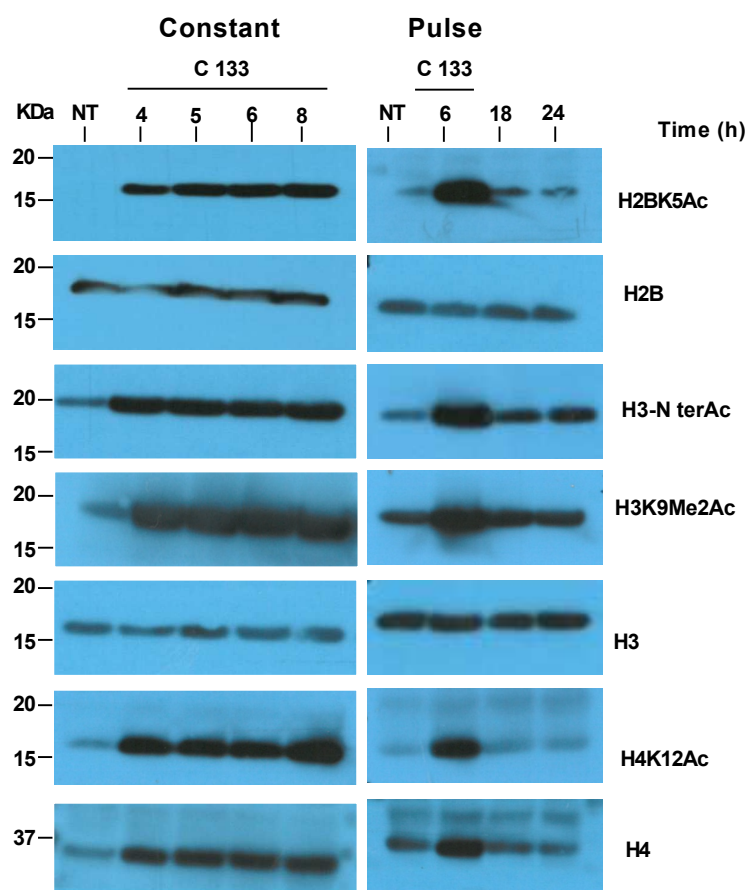

**B**

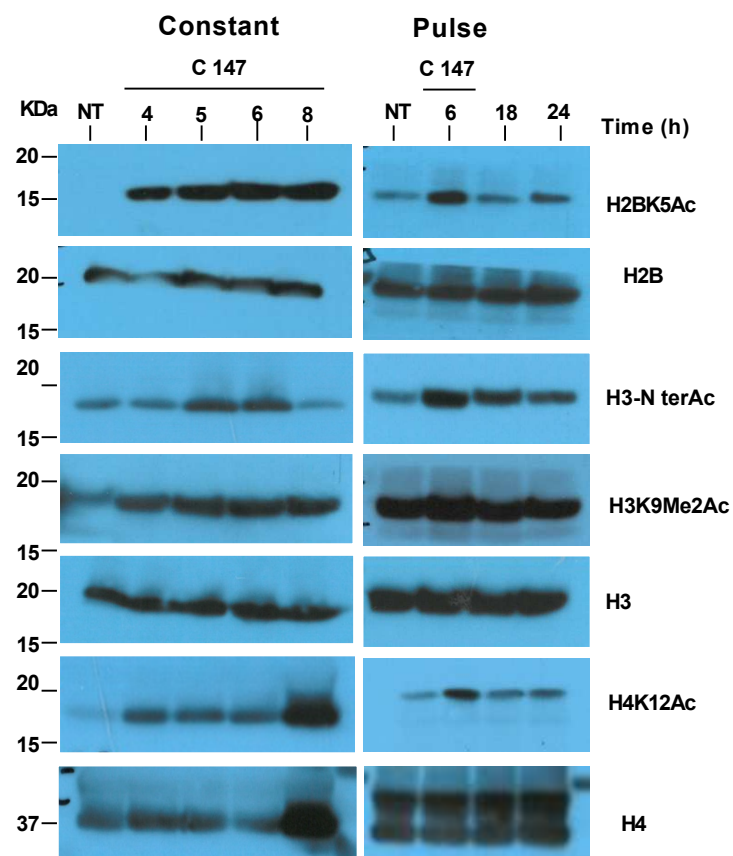

Figure 7S

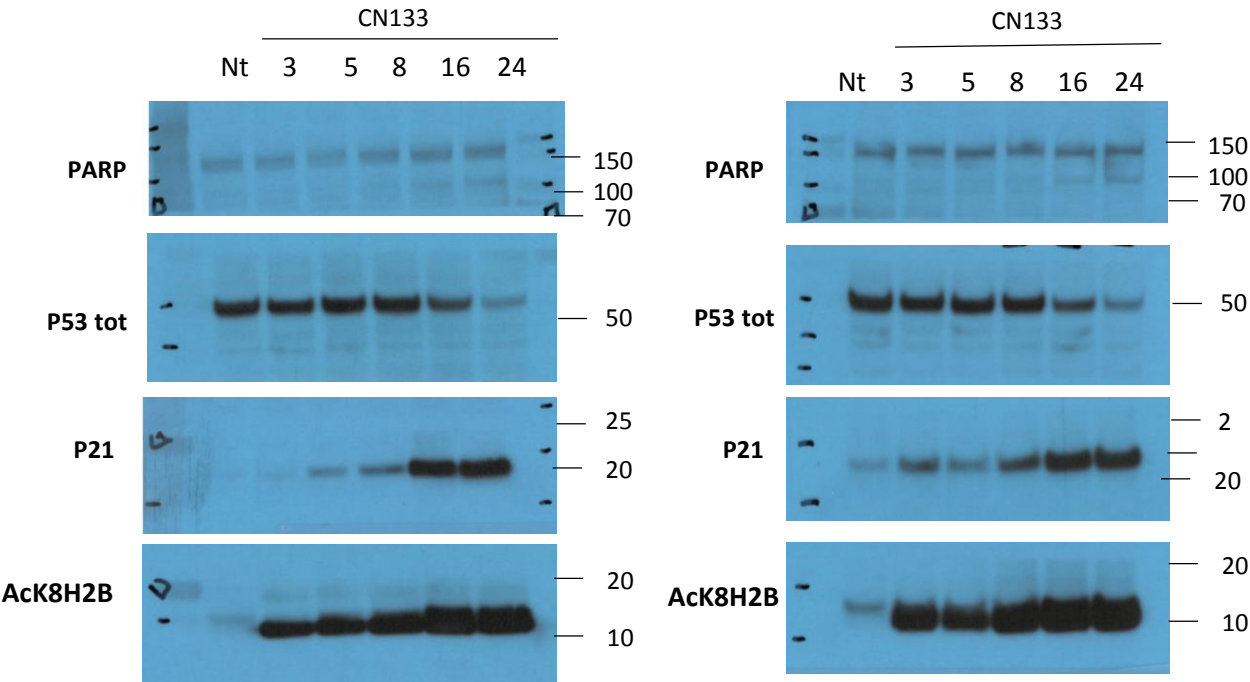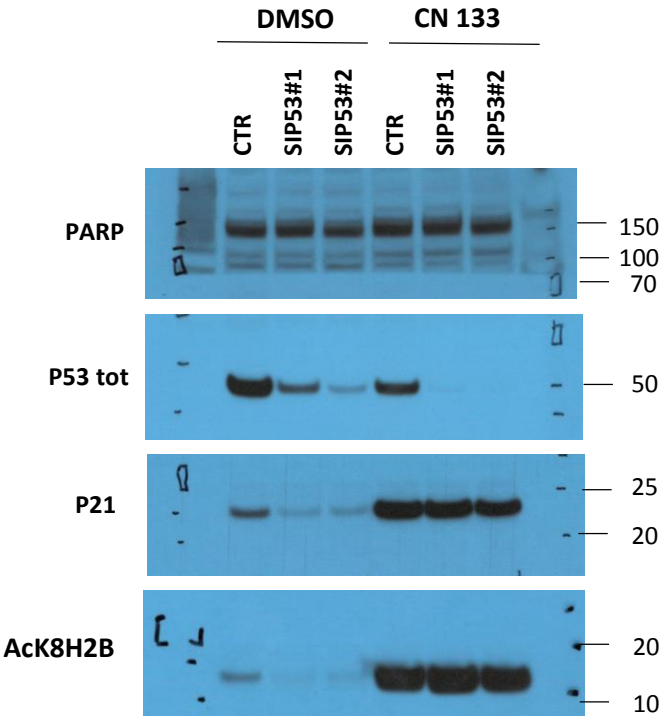

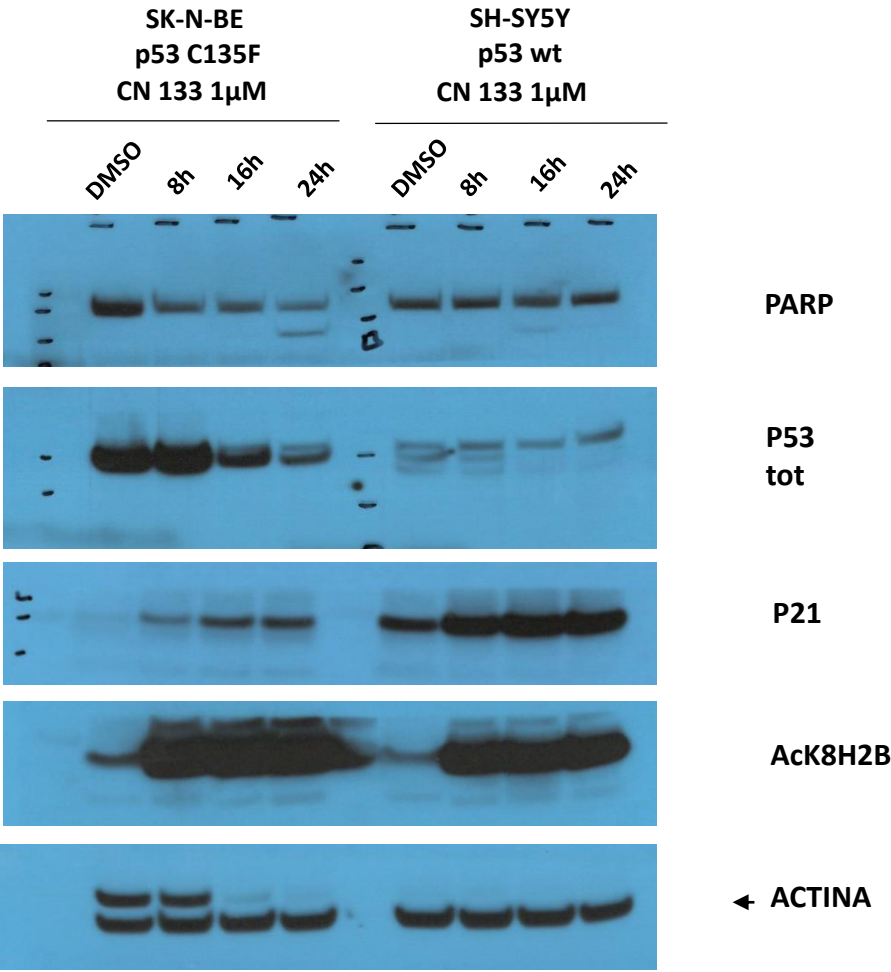

Supplement: Supplementary file 1 [file cancers-12-01922-s001.zip › cancers-863779-supplementary final/Figure S9. Uncropped Western Blot Figures.pdf]
